# Bis(2,6-dihy­droxy­benzoato-κ^2^ *O* ^1^,*O* ^1′^)(nitrato-κ^2^ *O*,*O*′)bis­(1,10-phenanthroline-κ^2^ *N*,*N*′)praseodymium(III)

**DOI:** 10.1107/S1600536810049767

**Published:** 2010-12-04

**Authors:** Chiya Wang, Xiaojin Gu, Xinqing Wang, Hongxiao Jin

**Affiliations:** a College of Materials Science and Engineering, China Jiliang University, Hangzhou 310018, People’s Republic of China

## Abstract

The mononuclear title complex, [Pr(C_7_H_5_O_3_)_2_(NO_3_)(C_12_H_8_N_2_)_2_], is isostructural with related complexes of other lanthanides. The Pr(III) atom is in a pseudo-bicapped square-anti­prismatic geometry, formed by four N atoms from two chelating 1,10-phenanthroline (phen) ligands and six O atoms, four from two 2,6-dihy­droxy­benzoate (DHB) ligands and the other two from nitrate anions. π–π stacking inter­actions between the phen and DHB ligands [centroid–centroid distances = 3.518 (2) and 3.778 (2) Å] and the phen and phen ligands [face-to-face separation = 3.427 (6) Å] of adjacent complexes stabilize the crystal structure. Intra­molecular O—H⋯O hydrogen bonds are observed in the DHB ligands.

## Related literature

For the background and a related structure, see: Zheng *et al.* (2010 ▶).
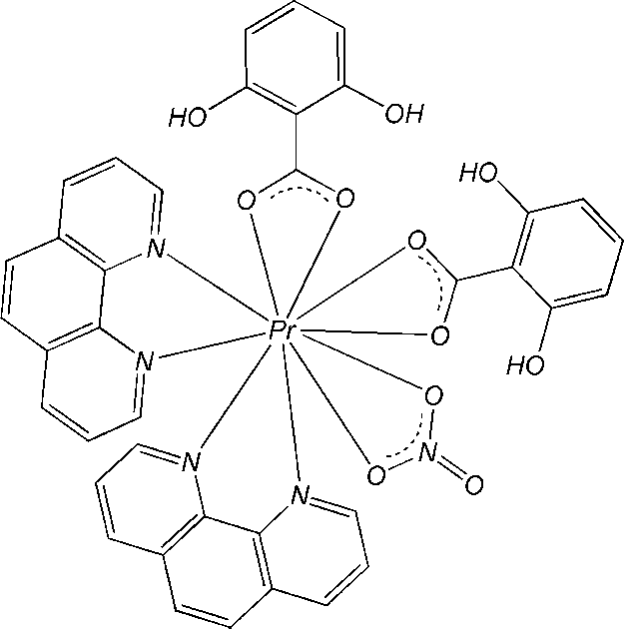


## Experimental

### 

#### Crystal data

[Pr(C_7_H_5_O_3_)_2_(NO_3_)(C_12_H_8_N_2_)_2_]
*M* *_r_* = 869.55
Monoclinic, 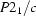

*a* = 11.2738 (2) Å
*b* = 26.8015 (5) Å
*c* = 14.3886 (4) Å
β = 127.934 (1)°
*V* = 3429.02 (13) Å^3^
*Z* = 4
Mo *K*α radiation
μ = 1.49 mm^−1^
*T* = 298 K
0.50 × 0.42 × 0.40 mm

#### Data collection

Oxford Diffraction Gemini S Ultra diffractometer
Absorption correction: multi-scan (*ABSPACK* in *CrysAlis PRO RED*; Oxford Diffraction, 2006 ▶) *T*_min_ = 0.522, *T*_max_ = 0.586
28864 measured reflections
6985 independent reflections
6611 reflections with *I* > 2σ(*I*)
*R*_int_ = 0.023

#### Refinement

*R*[*F* ^2^ > 2σ(*F* ^2^)] = 0.035
*wR*(*F* ^2^) = 0.075
*S* = 1.23
6985 reflections
500 parameters
H-atom parameters constrained
Δρ_max_ = 0.66 e Å^−3^
Δρ_min_ = −1.21 e Å^−3^

### 

Data collection: *CrysAlis PRO CCD* (Oxford Diffraction, 2006 ▶); cell refinement: *CrysAlis PRO CCD*; data reduction: *CrysAlis PRO RED* (Oxford Diffraction, 2006 ▶); program(s) used to solve structure: *SHELXS97* (Sheldrick, 2008 ▶); program(s) used to refine structure: *SHELXL97* (Sheldrick, 2008 ▶); molecular graphics: *DIAMOND* (Brandenburg & Berndt, 1999 ▶); software used to prepare material for publication: *SHELXL97*.

## Supplementary Material

Crystal structure: contains datablocks I, global. DOI: 10.1107/S1600536810049767/fj2366sup1.cif

Structure factors: contains datablocks I. DOI: 10.1107/S1600536810049767/fj2366Isup2.hkl

Additional supplementary materials:  crystallographic information; 3D view; checkCIF report

## Figures and Tables

**Table 1:** Hydrogen-bond geometry (Å, °)

*D*—H⋯*A*	*D*—H	H⋯*A*	*D*⋯*A*	*D*—H⋯*A*
O8—H38⋯O6	0.82	1.83	2.561 (4)	148
O7—H33⋯O5	0.82	1.87	2.592 (3)	147
O4—H31⋯O2	0.82	1.86	2.586 (4)	147
O3—H27⋯O1	0.82	1.85	2.577 (4)	147

**Fig. 1.**
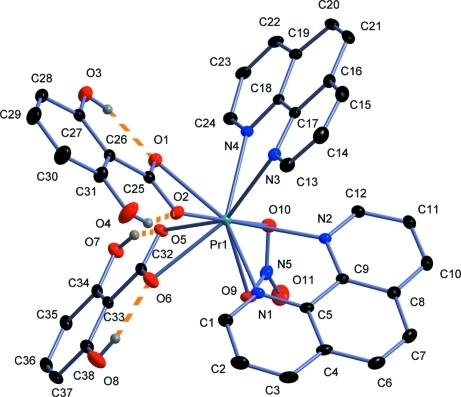
The molecular structure of title compound. Displacement ellipsoids are drawn at the 15% probablility level and H atoms are shown as small spheres of arbitraty radii. Some H atoms are omitted for clarity. Light orange lines show the intramolecular hygogen bonds.

## supplementary crystallographic information

### Comment

The description of the structure of the title compound is part of a series of papers on mononuclear complexes of the type [Ln(C_12_H_8_N_2_)_2_(C_7_H_8_O_3_)_2_ (NO_3_)], with Ln = Ce, Pr (this publication), Sm, Eu, and Dy respectively. All five compounds are also isostructural to the previously reported Nd complex (Zheng *et al.* 2010). The background to this study is given in previous paper by Zheng *et al*. (2010).

### Experimental

Each reagent was commercially available and of analytical grade. Pr(NO_3_)_3_^.^6H_2_O (0.217 g, 0.5 mmol), 2, 6-dihydroxybenzoic acid (0.074 g 0.5 mmol), 1, 10-phenanthroline (0.090 g, 0.5 mmol) and NaHCO_3_ (0.042 g, 0.5 mmol) were dissolved in water-ethanol solution (10 ml, 5:5). The solution was refluxed for 4 h, and filtered after cooling to room temperature. Yellow single crystals were obtained from the filtrate after 2 d.

### Refinement

H atoms were positioned geometrically (C—H = 0.93 Å and O—H = 0.82 Å) and refined as riding, with *U*_iso _(H) = 1.2Ueq (C) and *U*_iso_(H) = 1.5Ueq (O).

### Crystal data

**Table:**

[Pr(C_7_H_5_O_3_)_2_(NO_3_)(C_12_H_8_N_2_)_2_]	*F*(000) = 1744
*M**_r_* = 869.55	*D*_x_ = 1.684 Mg m^−^^3^
Monoclinic, *P*2_1_/*c*	Mo *K*α radiation, λ = 0.71073 Å
Hall symbol: -P 2ybc	Cell parameters from 18285 reflections
*a* = 11.2738 (2) Å	θ = 2.9–26.3°
*b* = 26.8015 (5) Å	µ = 1.49 mm^−^^1^
*c* = 14.3886 (4) Å	*T* = 298 K
β = 127.934 (1)°	Block, yellow
*V* = 3429.02 (13) Å^3^	0.50 × 0.42 × 0.40 mm
*Z* = 4	

### Data collection

**Table:**

Oxford Diffraction Gemini S Ultra diffractometer	6985 independent reflections
Radiation source: Enhance (Mo) X-ray Source	6611 reflections with *I* > 2σ(*I*)
graphite	*R*_int_ = 0.023
Detector resolution: 15.9149 pixels mm^-1^	θ_max_ = 26.4°, θ_min_ = 2.9°
ω scans	*h* = −14→13
Absorption correction: multi-scan (ABSPACK in *CrysAlis PRO* RED; Oxford Diffraction, 2006)	*k* = −33→33
*T*_min_ = 0.522, *T*_max_ = 0.586	*l* = −17→17
28864 measured reflections	

### Refinement

**Table:**

Refinement on *F*^2^	Primary atom site location: structure-invariant direct methods
Least-squares matrix: full	Secondary atom site location: difference Fourier map
*R*[*F*^2^ > 2σ(*F*^2^)] = 0.035	Hydrogen site location: inferred from neighbouring sites
*wR*(*F*^2^) = 0.075	H-atom parameters constrained
*S* = 1.23	*w* = 1/[σ^2^(*F*_o_^2^) + (0.0218*P*)^2^ + 4.6197*P*] where *P* = (*F*_o_^2^ + 2*F*_c_^2^)/3
6985 reflections	(Δ/σ)_max_ = 0.005
500 parameters	Δρ_max_ = 0.66 e Å^−^^3^
0 restraints	Δρ_min_ = −1.21 e Å^−^^3^

### Special details

**Table:**

Geometry. All e.s.d.'s (except the e.s.d. in the dihedral angle between two l.s. planes) are estimated using the full covariance matrix. The cell e.s.d.'s are taken into account individually in the estimation of e.s.d.'s in distances, angles and torsion angles; correlations between e.s.d.'s in cell parameters are only used when they are defined by crystal symmetry. An approximate (isotropic) treatment of cell e.s.d.'s is used for estimating e.s.d.'s involving l.s. planes.
Refinement. Refinement of *F*^2^ against ALL reflections. The weighted *R*-factor *wR* and goodness of fit *S* are based on *F*^2^, conventional *R*-factors *R* are based on *F*, with *F* set to zero for negative *F*^2^. The threshold expression of *F*^2^ > σ(*F*^2^) is used only for calculating *R*-factors(gt) *etc*. and is not relevant to the choice of reflections for refinement. *R*-factors based on *F*^2^ are statistically about twice as large as those based on *F*, and *R*- factors based on ALL data will be even larger.

### Fractional atomic coordinates and isotropic or equivalent isotropic displacement parameters (Å^2^)

**Table:**

	*x*	*y*	*z*	*U*_iso_*/*U*_eq_	
Pr1	0.431931 (19)	0.861161 (6)	0.219170 (15)	0.02837 (6)	
O1	0.3448 (3)	0.83800 (9)	0.0146 (2)	0.0418 (6)	
O2	0.3881 (3)	0.91821 (9)	0.0538 (2)	0.0412 (6)	
O3	0.2586 (4)	0.80310 (10)	−0.1851 (2)	0.0563 (7)	
H27	0.2842	0.8022	−0.1180	0.084*	
O4	0.3341 (5)	0.97961 (11)	−0.1068 (3)	0.0805 (11)	
H31	0.3535	0.9709	−0.0441	0.121*	
O5	0.1925 (3)	0.81083 (9)	0.1310 (2)	0.0408 (6)	
O6	0.1610 (3)	0.89160 (10)	0.0956 (3)	0.0499 (7)	
O7	−0.0202 (3)	0.75706 (10)	0.1003 (3)	0.0491 (6)	
H33	0.0631	0.7630	0.1187	0.074*	
O8	−0.0826 (4)	0.93463 (11)	0.0293 (3)	0.0704 (9)	
H38	0.0025	0.9320	0.0489	0.106*	
O9	0.3498 (3)	0.86921 (10)	0.3507 (3)	0.0495 (7)	
O10	0.4607 (3)	0.79976 (9)	0.3731 (2)	0.0432 (6)	
O11	0.3690 (4)	0.81302 (16)	0.4668 (3)	0.0820 (11)	
N1	0.4773 (3)	0.95579 (10)	0.2922 (2)	0.0336 (6)	
N2	0.6704 (3)	0.88251 (10)	0.4412 (2)	0.0321 (6)	
N3	0.6879 (3)	0.86563 (10)	0.2479 (2)	0.0350 (6)	
N4	0.5639 (3)	0.77687 (10)	0.2377 (2)	0.0326 (6)	
N5	0.3932 (3)	0.82624 (13)	0.3990 (3)	0.0438 (7)	
C1	0.3829 (4)	0.99163 (13)	0.2206 (3)	0.0431 (8)	
H1	0.3078	0.9838	0.1419	0.052*	
C2	0.3913 (5)	1.04074 (14)	0.2584 (4)	0.0523 (10)	
H2	0.3233	1.0648	0.2054	0.063*	
C3	0.4994 (5)	1.05269 (13)	0.3727 (4)	0.0481 (10)	
H3	0.5056	1.0850	0.3989	0.058*	
C4	0.6020 (4)	1.01638 (12)	0.4516 (3)	0.0381 (8)	
C5	0.5852 (4)	0.96780 (11)	0.4067 (3)	0.0312 (7)	
C6	0.7189 (5)	1.02637 (14)	0.5732 (4)	0.0480 (10)	
H6	0.7291	1.0584	0.6023	0.058*	
C7	0.8144 (5)	0.99044 (15)	0.6463 (4)	0.0481 (9)	
H7	0.8895	0.9980	0.7252	0.058*	
C8	0.8032 (4)	0.94058 (14)	0.6054 (3)	0.0383 (8)	
C9	0.6881 (4)	0.92924 (12)	0.4856 (3)	0.0308 (7)	
C10	0.9003 (4)	0.90184 (16)	0.6780 (3)	0.0460 (9)	
H10	0.9763	0.9078	0.7576	0.055*	
C11	0.8840 (4)	0.85562 (15)	0.6324 (3)	0.0439 (9)	
H11	0.9493	0.8299	0.6797	0.053*	
C12	0.7675 (4)	0.84786 (13)	0.5136 (3)	0.0383 (8)	
H12	0.7572	0.8162	0.4831	0.046*	
C13	0.7477 (4)	0.90873 (14)	0.2511 (3)	0.0448 (9)	
H13	0.6896	0.9375	0.2278	0.054*	
C14	0.8952 (5)	0.91295 (17)	0.2881 (4)	0.0541 (10)	
H14	0.9332	0.9438	0.2882	0.065*	
C15	0.9818 (5)	0.87108 (18)	0.3239 (4)	0.0546 (11)	
H15	1.0806	0.8734	0.3506	0.066*	
C16	0.9230 (4)	0.82471 (16)	0.3206 (3)	0.0430 (9)	
C17	0.7734 (4)	0.82384 (13)	0.2813 (3)	0.0333 (7)	
C18	0.7056 (4)	0.77663 (12)	0.2710 (3)	0.0313 (7)	
C19	0.7863 (4)	0.73205 (14)	0.2954 (3)	0.0396 (8)	
C20	0.9393 (5)	0.73500 (17)	0.3391 (3)	0.0510 (10)	
H20	0.9946	0.7058	0.3591	0.061*	
C21	1.0047 (4)	0.77876 (18)	0.3520 (3)	0.0537 (11)	
H21	1.1049	0.7794	0.3818	0.064*	
C22	0.7104 (5)	0.68711 (13)	0.2759 (3)	0.0467 (9)	
H22	0.7584	0.6568	0.2880	0.056*	
C23	0.5679 (5)	0.68766 (13)	0.2395 (3)	0.0464 (9)	
H23	0.5168	0.6579	0.2254	0.056*	
C24	0.4982 (4)	0.73345 (12)	0.2232 (3)	0.0394 (8)	
H24	0.4015	0.7334	0.2012	0.047*	
C25	0.3461 (4)	0.88195 (13)	−0.0172 (3)	0.0343 (7)	
C26	0.2987 (4)	0.89075 (13)	−0.1371 (3)	0.0333 (7)	
C27	0.2560 (4)	0.85057 (13)	−0.2155 (3)	0.0382 (8)	
C28	0.2090 (4)	0.85953 (17)	−0.3286 (3)	0.0511 (10)	
H28	0.1809	0.8331	−0.3802	0.061*	
C29	0.2040 (5)	0.90735 (18)	−0.3640 (4)	0.0578 (11)	
H29	0.1723	0.9129	−0.4400	0.069*	
C30	0.2444 (6)	0.94733 (17)	−0.2904 (4)	0.0612 (12)	
H30	0.2386	0.9796	−0.3169	0.073*	
C31	0.2939 (5)	0.93947 (15)	−0.1767 (3)	0.0483 (9)	
C32	0.1106 (4)	0.84976 (13)	0.0979 (3)	0.0374 (8)	
C33	−0.0420 (4)	0.84599 (13)	0.0656 (3)	0.0357 (7)	
C34	−0.0992 (4)	0.79969 (14)	0.0687 (3)	0.0384 (8)	
C35	−0.2423 (4)	0.79683 (17)	0.0385 (3)	0.0462 (9)	
H35	−0.2804	0.7664	0.0403	0.055*	
C36	−0.3263 (4)	0.83955 (19)	0.0062 (3)	0.0544 (11)	
H36	−0.4219	0.8374	−0.0140	0.065*	
C37	−0.2749 (4)	0.88508 (18)	0.0026 (4)	0.0556 (11)	
H37	−0.3349	0.9133	−0.0199	0.067*	
C38	−0.1327 (4)	0.88879 (15)	0.0326 (3)	0.0470 (9)	

### Atomic displacement parameters (Å^2^)

**Table:**

	*U*^11^	*U*^22^	*U*^33^	*U*^12^	*U*^13^	*U*^23^
Pr1	0.03009 (10)	0.02348 (9)	0.03471 (10)	0.00047 (7)	0.02153 (8)	−0.00019 (7)
O1	0.0506 (15)	0.0316 (13)	0.0398 (13)	−0.0010 (11)	0.0260 (13)	0.0012 (10)
O2	0.0526 (15)	0.0350 (13)	0.0384 (13)	−0.0035 (11)	0.0291 (13)	−0.0021 (11)
O3	0.0658 (19)	0.0384 (15)	0.0455 (16)	0.0007 (13)	0.0245 (16)	−0.0046 (12)
O4	0.145 (3)	0.0380 (16)	0.0548 (19)	−0.0181 (19)	0.060 (2)	−0.0043 (14)
O5	0.0312 (13)	0.0359 (13)	0.0541 (15)	0.0018 (10)	0.0255 (12)	−0.0002 (11)
O6	0.0384 (14)	0.0397 (14)	0.0680 (18)	0.0035 (11)	0.0309 (14)	0.0124 (13)
O7	0.0402 (15)	0.0440 (15)	0.0648 (18)	−0.0003 (11)	0.0331 (15)	0.0062 (13)
O8	0.0528 (19)	0.0512 (18)	0.097 (3)	0.0174 (14)	0.041 (2)	0.0164 (17)
O9	0.0594 (17)	0.0426 (15)	0.0621 (17)	0.0034 (12)	0.0452 (16)	−0.0062 (13)
O10	0.0492 (15)	0.0353 (13)	0.0512 (15)	0.0001 (11)	0.0339 (14)	0.0040 (11)
O11	0.080 (2)	0.130 (3)	0.0579 (19)	−0.004 (2)	0.053 (2)	0.014 (2)
N1	0.0397 (16)	0.0260 (13)	0.0405 (16)	0.0030 (11)	0.0275 (14)	0.0024 (11)
N2	0.0361 (15)	0.0279 (13)	0.0359 (14)	0.0033 (11)	0.0239 (13)	0.0001 (11)
N3	0.0370 (15)	0.0347 (15)	0.0382 (15)	−0.0068 (12)	0.0256 (14)	−0.0042 (12)
N4	0.0366 (15)	0.0267 (13)	0.0370 (15)	0.0009 (11)	0.0239 (13)	−0.0011 (11)
N5	0.0417 (18)	0.056 (2)	0.0371 (16)	−0.0116 (15)	0.0259 (15)	−0.0062 (14)
C1	0.052 (2)	0.0325 (18)	0.048 (2)	0.0077 (16)	0.0322 (19)	0.0068 (16)
C2	0.070 (3)	0.033 (2)	0.071 (3)	0.0123 (18)	0.052 (3)	0.0126 (19)
C3	0.072 (3)	0.0224 (17)	0.077 (3)	−0.0008 (17)	0.060 (3)	−0.0007 (17)
C4	0.052 (2)	0.0240 (16)	0.061 (2)	−0.0086 (14)	0.047 (2)	−0.0071 (15)
C5	0.0389 (18)	0.0241 (15)	0.0454 (19)	−0.0040 (13)	0.0334 (17)	−0.0021 (13)
C6	0.059 (2)	0.0352 (19)	0.069 (3)	−0.0190 (18)	0.049 (2)	−0.0199 (19)
C7	0.048 (2)	0.052 (2)	0.052 (2)	−0.0209 (19)	0.034 (2)	−0.0213 (19)
C8	0.0342 (18)	0.045 (2)	0.045 (2)	−0.0099 (15)	0.0291 (17)	−0.0084 (16)
C9	0.0318 (17)	0.0300 (16)	0.0412 (18)	−0.0031 (13)	0.0277 (16)	−0.0027 (13)
C10	0.0309 (19)	0.064 (3)	0.0381 (19)	−0.0055 (17)	0.0187 (17)	−0.0054 (18)
C11	0.0350 (19)	0.052 (2)	0.042 (2)	0.0072 (16)	0.0225 (17)	0.0063 (17)
C12	0.0385 (19)	0.0374 (18)	0.0414 (19)	0.0060 (14)	0.0258 (17)	0.0038 (15)
C13	0.052 (2)	0.040 (2)	0.050 (2)	−0.0100 (17)	0.035 (2)	−0.0045 (16)
C14	0.054 (3)	0.058 (3)	0.058 (2)	−0.027 (2)	0.039 (2)	−0.013 (2)
C15	0.037 (2)	0.083 (3)	0.047 (2)	−0.014 (2)	0.028 (2)	−0.010 (2)
C16	0.0339 (19)	0.066 (3)	0.0331 (18)	−0.0043 (17)	0.0225 (17)	−0.0065 (17)
C17	0.0335 (18)	0.0430 (19)	0.0280 (16)	−0.0008 (14)	0.0212 (15)	−0.0030 (14)
C18	0.0346 (17)	0.0341 (17)	0.0271 (15)	0.0049 (13)	0.0199 (15)	−0.0005 (13)
C19	0.047 (2)	0.045 (2)	0.0304 (17)	0.0140 (16)	0.0258 (17)	0.0041 (15)
C20	0.049 (2)	0.064 (3)	0.043 (2)	0.024 (2)	0.029 (2)	0.0064 (19)
C21	0.033 (2)	0.085 (3)	0.042 (2)	0.016 (2)	0.0219 (18)	0.002 (2)
C22	0.070 (3)	0.0305 (18)	0.044 (2)	0.0152 (17)	0.037 (2)	0.0032 (15)
C23	0.066 (3)	0.0278 (18)	0.049 (2)	0.0023 (17)	0.037 (2)	−0.0024 (15)
C24	0.044 (2)	0.0289 (17)	0.047 (2)	−0.0015 (15)	0.0286 (18)	−0.0022 (15)
C25	0.0275 (17)	0.0393 (18)	0.0350 (17)	0.0000 (14)	0.0186 (15)	0.0006 (14)
C26	0.0266 (16)	0.0375 (18)	0.0333 (17)	−0.0019 (13)	0.0173 (15)	−0.0013 (14)
C27	0.0269 (17)	0.042 (2)	0.0391 (19)	0.0002 (14)	0.0170 (16)	−0.0034 (15)
C28	0.043 (2)	0.067 (3)	0.039 (2)	0.0001 (19)	0.0234 (19)	−0.0097 (19)
C29	0.065 (3)	0.072 (3)	0.040 (2)	−0.012 (2)	0.034 (2)	−0.002 (2)
C30	0.084 (3)	0.055 (3)	0.048 (2)	−0.013 (2)	0.043 (3)	0.005 (2)
C31	0.058 (3)	0.045 (2)	0.041 (2)	−0.0086 (18)	0.031 (2)	−0.0009 (17)
C32	0.0316 (18)	0.042 (2)	0.0318 (17)	0.0048 (14)	0.0160 (15)	0.0016 (14)
C33	0.0274 (17)	0.047 (2)	0.0305 (17)	0.0041 (14)	0.0166 (15)	0.0006 (14)
C34	0.0292 (17)	0.053 (2)	0.0297 (17)	0.0000 (15)	0.0163 (15)	0.0006 (15)
C35	0.0327 (19)	0.071 (3)	0.0373 (19)	−0.0031 (18)	0.0227 (17)	0.0006 (18)
C36	0.030 (2)	0.094 (3)	0.041 (2)	0.003 (2)	0.0231 (18)	−0.001 (2)
C37	0.039 (2)	0.075 (3)	0.050 (2)	0.022 (2)	0.027 (2)	0.008 (2)
C38	0.041 (2)	0.051 (2)	0.045 (2)	0.0077 (17)	0.0239 (19)	0.0021 (17)

### Geometric parameters (Å, °)

**Table:**

Pr1—O1	2.542 (2)	C8—C9	1.414 (5)
Pr1—O6	2.546 (2)	C10—C11	1.360 (5)
Pr1—O5	2.551 (2)	C10—H10	0.9300
Pr1—O9	2.577 (3)	C11—C12	1.390 (5)
Pr1—O2	2.607 (2)	C11—H11	0.9300
Pr1—O10	2.615 (2)	C12—H12	0.9300
Pr1—N4	2.627 (3)	C13—C14	1.404 (5)
Pr1—N3	2.654 (3)	C13—H13	0.9300
Pr1—N1	2.672 (3)	C14—C15	1.364 (6)
Pr1—N2	2.683 (3)	C14—H14	0.9300
O1—C25	1.267 (4)	C15—C16	1.396 (6)
O2—C25	1.272 (4)	C15—H15	0.9300
O3—C27	1.340 (4)	C16—C17	1.411 (5)
O3—H27	0.8200	C16—C21	1.435 (6)
O4—C31	1.347 (5)	C17—C18	1.438 (5)
O4—H31	0.8200	C18—C19	1.409 (4)
O5—C32	1.276 (4)	C19—C22	1.402 (5)
O6—C32	1.267 (4)	C19—C20	1.429 (5)
O7—C34	1.344 (4)	C20—C21	1.336 (6)
O7—H33	0.8200	C20—H20	0.9300
O8—C38	1.365 (5)	C21—H21	0.9300
O8—H38	0.8200	C22—C23	1.349 (6)
O9—N5	1.277 (4)	C22—H22	0.9300
O10—N5	1.253 (4)	C23—C24	1.397 (5)
O11—N5	1.216 (4)	C23—H23	0.9300
N1—C1	1.330 (4)	C24—H24	0.9300
N1—C5	1.355 (4)	C25—C26	1.478 (5)
N2—C12	1.321 (4)	C26—C31	1.412 (5)
N2—C9	1.364 (4)	C26—C27	1.413 (5)
N3—C13	1.324 (4)	C27—C28	1.387 (5)
N3—C17	1.358 (4)	C28—C29	1.368 (6)
N4—C24	1.325 (4)	C28—H28	0.9300
N4—C18	1.357 (4)	C29—C30	1.373 (6)
C1—C2	1.405 (5)	C29—H29	0.9300
C1—H1	0.9300	C30—C31	1.382 (5)
C2—C3	1.353 (6)	C30—H30	0.9300
C2—H2	0.9300	C32—C33	1.484 (5)
C3—C4	1.400 (5)	C33—C38	1.411 (5)
C3—H3	0.9300	C33—C34	1.412 (5)
C4—C5	1.413 (4)	C34—C35	1.392 (5)
C4—C6	1.426 (5)	C35—C36	1.372 (6)
C5—C9	1.443 (5)	C35—H35	0.9300
C6—C7	1.342 (6)	C36—C37	1.366 (6)
C6—H6	0.9300	C36—H36	0.9300
C7—C8	1.434 (5)	C37—C38	1.384 (5)
C7—H7	0.9300	C37—H37	0.9300
C8—C10	1.401 (5)		
			
O1—Pr1—O6	79.96 (9)	C10—C8—C7	123.6 (4)
O1—Pr1—O5	75.95 (8)	C9—C8—C7	119.0 (3)
O6—Pr1—O5	51.21 (8)	N2—C9—C8	122.0 (3)
O1—Pr1—O9	144.43 (9)	N2—C9—C5	118.3 (3)
O6—Pr1—O9	70.59 (9)	C8—C9—C5	119.7 (3)
O5—Pr1—O9	70.21 (8)	C11—C10—C8	120.3 (3)
O1—Pr1—O2	50.56 (7)	C11—C10—H10	119.9
O6—Pr1—O2	72.64 (8)	C8—C10—H10	119.9
O5—Pr1—O2	107.89 (8)	C10—C11—C12	118.3 (4)
O9—Pr1—O2	132.00 (8)	C10—C11—H11	120.8
O1—Pr1—O10	125.86 (8)	C12—C11—H11	120.8
O6—Pr1—O10	105.32 (8)	N2—C12—C11	124.3 (3)
O5—Pr1—O10	68.33 (8)	N2—C12—H12	117.8
O9—Pr1—O10	48.99 (8)	C11—C12—H12	117.8
O2—Pr1—O10	175.89 (8)	N3—C13—C14	122.8 (4)
O1—Pr1—N4	72.48 (8)	N3—C13—H13	118.6
O6—Pr1—N4	135.07 (9)	C14—C13—H13	118.6
O5—Pr1—N4	87.46 (8)	C15—C14—C13	118.9 (4)
O9—Pr1—N4	115.74 (8)	C15—C14—H14	120.6
O2—Pr1—N4	112.00 (8)	C13—C14—H14	120.6
O10—Pr1—N4	66.75 (8)	C14—C15—C16	120.3 (4)
O1—Pr1—N3	78.50 (8)	C14—C15—H15	119.8
O6—Pr1—N3	144.80 (9)	C16—C15—H15	119.8
O5—Pr1—N3	144.90 (8)	C15—C16—C17	117.0 (4)
O9—Pr1—N3	136.79 (9)	C15—C16—C21	123.6 (4)
O2—Pr1—N3	72.16 (8)	C17—C16—C21	119.4 (4)
O10—Pr1—N3	109.85 (8)	N3—C17—C16	122.8 (3)
N4—Pr1—N3	61.92 (8)	N3—C17—C18	118.2 (3)
O1—Pr1—N1	122.12 (8)	C16—C17—C18	118.9 (3)
O6—Pr1—N1	80.08 (9)	N4—C18—C19	122.1 (3)
O5—Pr1—N1	125.86 (8)	N4—C18—C17	117.9 (3)
O9—Pr1—N1	72.53 (8)	C19—C18—C17	120.0 (3)
O2—Pr1—N1	71.67 (8)	C22—C19—C18	117.2 (3)
O10—Pr1—N1	111.71 (8)	C22—C19—C20	123.9 (3)
N4—Pr1—N1	144.79 (9)	C18—C19—C20	118.8 (4)
N3—Pr1—N1	88.23 (8)	C21—C20—C19	121.6 (4)
O1—Pr1—N2	145.28 (8)	C21—C20—H20	119.2
O6—Pr1—N2	131.08 (9)	C19—C20—H20	119.2
O5—Pr1—N2	133.19 (8)	C20—C21—C16	121.1 (4)
O9—Pr1—N2	69.95 (9)	C20—C21—H21	119.4
O2—Pr1—N2	116.77 (8)	C16—C21—H21	119.4
O10—Pr1—N2	67.29 (8)	C23—C22—C19	120.2 (3)
N4—Pr1—N2	88.41 (8)	C23—C22—H22	119.9
N3—Pr1—N2	66.88 (8)	C19—C22—H22	119.9
N1—Pr1—N2	61.30 (8)	C22—C23—C24	119.2 (4)
C25—O1—Pr1	96.3 (2)	C22—C23—H23	120.4
C25—O2—Pr1	93.05 (19)	C24—C23—H23	120.4
C27—O3—H27	109.5	N4—C24—C23	122.8 (3)
C31—O4—H31	109.5	N4—C24—H24	118.6
C32—O5—Pr1	93.1 (2)	C23—C24—H24	118.6
C32—O6—Pr1	93.6 (2)	O1—C25—O2	120.1 (3)
C34—O7—H33	109.5	O1—C25—C26	119.6 (3)
C38—O8—H38	109.5	O2—C25—C26	120.3 (3)
N5—O9—Pr1	97.69 (19)	C31—C26—C27	118.2 (3)
N5—O10—Pr1	96.53 (19)	C31—C26—C25	121.0 (3)
C1—N1—C5	117.6 (3)	C27—C26—C25	120.8 (3)
C1—N1—Pr1	120.8 (2)	O3—C27—C28	117.6 (3)
C5—N1—Pr1	121.0 (2)	O3—C27—C26	122.3 (3)
C12—N2—C9	117.7 (3)	C28—C27—C26	120.1 (3)
C12—N2—Pr1	121.8 (2)	C29—C28—C27	119.9 (4)
C9—N2—Pr1	120.3 (2)	C29—C28—H28	120.1
C13—N3—C17	118.1 (3)	C27—C28—H28	120.1
C13—N3—Pr1	121.8 (2)	C28—C29—C30	121.7 (4)
C17—N3—Pr1	119.0 (2)	C28—C29—H29	119.1
C24—N4—C18	118.3 (3)	C30—C29—H29	119.1
C24—N4—Pr1	120.8 (2)	C29—C30—C31	119.7 (4)
C18—N4—Pr1	120.7 (2)	C29—C30—H30	120.2
O11—N5—O10	123.4 (4)	C31—C30—H30	120.2
O11—N5—O9	120.0 (4)	O4—C31—C30	117.9 (4)
O10—N5—O9	116.6 (3)	O4—C31—C26	121.7 (3)
N1—C1—C2	123.1 (4)	C30—C31—C26	120.4 (4)
N1—C1—H1	118.5	O6—C32—O5	120.1 (3)
C2—C1—H1	118.5	O6—C32—C33	120.3 (3)
C3—C2—C1	119.2 (4)	O5—C32—C33	119.6 (3)
C3—C2—H2	120.4	C38—C33—C34	118.3 (3)
C1—C2—H2	120.4	C38—C33—C32	120.8 (3)
C2—C3—C4	120.0 (3)	C34—C33—C32	120.9 (3)
C2—C3—H3	120.0	O7—C34—C35	117.3 (3)
C4—C3—H3	120.0	O7—C34—C33	122.5 (3)
C3—C4—C5	117.3 (3)	C35—C34—C33	120.2 (3)
C3—C4—C6	123.0 (3)	C36—C35—C34	119.1 (4)
C5—C4—C6	119.8 (3)	C36—C35—H35	120.5
N1—C5—C4	122.9 (3)	C34—C35—H35	120.5
N1—C5—C9	118.2 (3)	C37—C36—C35	122.5 (4)
C4—C5—C9	118.9 (3)	C37—C36—H36	118.7
C7—C6—C4	121.2 (3)	C35—C36—H36	118.7
C7—C6—H6	119.4	C36—C37—C38	119.3 (4)
C4—C6—H6	119.4	C36—C37—H37	120.3
C6—C7—C8	121.4 (4)	C38—C37—H37	120.3
C6—C7—H7	119.3	O8—C38—C37	118.6 (4)
C8—C7—H7	119.3	O8—C38—C33	120.9 (3)
C10—C8—C9	117.4 (3)	C37—C38—C33	120.5 (4)

### Hydrogen-bond geometry (Å, °)

**Table:**

*D*—H···*A*	*D*—H	H···*A*	*D*···*A*	*D*—H···*A*
O8—H38···O6	0.82	1.83	2.561 (4)	148
O7—H33···O5	0.82	1.87	2.592 (3)	147
O4—H31···O2	0.82	1.86	2.586 (4)	147
O3—H27···O1	0.82	1.85	2.577 (4)	147

## References

[bb1] Brandenburg, K. & Berndt, M. (1999). *DIAMOND* Crystal Impact GbR, Bonn, Germany.

[bb2] Oxford Diffraction (2006). *CrysAlis PRO CCD* and *CrysAlis PRO RED* Oxford Diffraction Ltd, Abingdon, England.

[bb3] Sheldrick, G. M. (2008). *Acta Cryst.* A**64**, 112–122. 10.1107/S0108767307043930 18156677

[bb4] Zheng, J., Jin, H. & Ge, H. (2010). *Acta Cryst.* E**66**, m1469–m1470. 10.1107/S1600536810042583 PMC3009246 21588886

